# Increasing the Yield of Irish Brown Crab (*Cancer pagurus*) during Processing without Adversely Affecting Shelf-Life

**DOI:** 10.3390/foods7070099

**Published:** 2018-06-27

**Authors:** Aoife McDermott, Paul Whyte, Nigel Brunton, James Lyng, Declan J. Bolton

**Affiliations:** 1 Teagasc Food Research Centre, Ashtown, Dublin 15, Ireland; aoife.mcdermott@teagasc.ie; 2 School of Veterinary Medicine, University College Dublin, Belfield, Dublin 4, Ireland; paul.whyte@ucd.ie; 3 School of Agriculture and Food Science, University College Dublin, Belfield, Dublin 4, Ireland; nigel.brunton@ucd.ie (N.B.); james.lyng@ucd.ie (J.L.)

**Keywords:** seafood, crustaceans, processing yield, shelf life, spoilage bacteria

## Abstract

During the processing of Irish Brown Crab (*Cancer pagurus*), protein and moisture are released and losses up to 10% (by weight) are common. The objective of this study was to investigate the use of clean label ingredients to reduce this loss, without adversely affecting shelf-life or promoting the growth of spoilage bacteria. Following preliminary studies, 5% (*w/v*) sodium caseinate (SC) and (5%, *w/v*) potato starch (PS), with and without (0.5%, *w/v*) ascorbic acid (AA) were selected. Ninety crabs (30 per treatment) were soaked and boiled in water (control 1), AA (control 2), SC, PS, SC plus AA, or PS plus AA and analyzed for cook loss as well as pH, aw, water holding capacity (WHC), and microbial shelf-life (total viable count (TVC), total Enterobacteriaceae count (TEC), and spoilage bacteria) during 28 days storage at 4 °C. On average, 11.1% of the control 1 weight was lost during processing. This was reduced to 8.0% when treated with AA (control 2) and to 3.5%, 4.7%, 5.8%, and 2.3% with SC, PS, SC plus AA, and PS plus AA, respectively. None of these treatments negatively impacted on shelf-life and similar growth curves were observed for TVC, TEC, *Pseudomonas* spp., *Clostridium* spp., lactic acid bacteria (LAB), and hydrogen disulphide producing bacteria, regardless of treatment. It was therefore concluded that, subject to sensory evaluation and validation under commercial conditions, these natural ingredients could be used to substantially increase the yield and hence commercial value of crab meat, without adversely affecting shelf-life.

## 1. Introduction

The Irish Brown crab, also referred to as the ‘edible crab’ or ‘common crab’ (*Cancer pagurus*) is a commercially important decapod species in Europe. With approximately 6000 tons landed annually in Ireland, this industry is worth in excess of €43 m to the Irish seafood sector [1]. Indeed, on a global scale, Ireland is one of the top three producers of brown crab products [2]. Traditionally crab is processed whole and vacuum packaged but current trends are moving towards picked meat, thereby adding value and providing consumers with a more convenient product that can be used in a range of dishes/meals [3]. Post euthanization, crabs are typically soaked in water at ambient temperature for 2 h, and boiled at 100 °C for 20 min, at which stage the meat may be extracted or the crab may remain intact, depending on the final product. A cook loss of typically 10% after boiling is common [4]. Products are then vacuum packed followed by pasteurization (typically 70 °C for 2 min (picked meat) or 90 °C for 10 min (whole crab)). 

Functional ingredients, such as binding agents, bind with water, thereby preventing cook loss and generally increasing meat quality [5]. Phosphates, which were traditionally used in a range of processed foods, including seafood, are no longer acceptable to consumers who prefer ‘natural’ ingredients that do not have a negative environmental impact [6,7]. Natural alternatives include casein and starch [8,9,10,11]. 

Sodium caseinate is a protein derived from bovine milk that binds fat with water, thereby increasing yield [12]. It can also bind with proteins in the crab, minimizing exudate and preventing cook loss [12] as has been observed with cooked hams [13]. Potato starch, although commonly used as a gelling agent and water binder [14], has, to the best of our knowledge, never been used in seafood and crustacean products.

However, the incorporation of protein and carbohydrate into the processing stages of crab may increase the nutrient available to bacteria thus promoting growth and increasing spoilage rates. Crab meat spoilage is predominately due to the activity of bacteria such as *Pseudomonas* spp., *Clostridium* spp., lactic acid bacteria (LAB), and hydrogen disulphide producing bacteria. This could be prevented by including a preservative such as ascorbic acid (vitamin C), which has ‘generally regarded as safe’ (GRAS) status for use in foods [15]. Moreover, it is particularly suitable for use in crab-based food products as it is naturally present in crab meat [16] and has been previously used to preserve seafood [17,18]. The objective of this study is to investigate the application of sodium caseinate and potato starch (with and without the preservative ascorbic acid) to increase the yield without adversely affecting the shelf-life of Irish brown crab. 

## 2. Materials and Method 

### 2.1. Sample Preparation 

Ninety (30 on 3 separate occasions) Irish brown crabs (*Cancer pagurus*) were obtained and euthanized by a local fishmonger. Crabs were then soaked for 2 h in 5% *w/v* sodium caseinate or 5% *w/v* potato starch, with and without 0.5% *w/v* ascorbic acid. Immersion in sterile distilled water (SDW) was used as a control (control 1), as was immersion in 0.5% *w/v* ascorbic acid (control 2). Crabs were weighed before and after soaking, and cooked in freshly prepared ingredient solutions for 20 min at 100 °C. The final cooked weight was recorded and crabs were allowed to cool at room temperature. Yield (%) after cooking was calculated using the formula of yield = weight after cooking/weight before cooking × 100.

White crab meat was handpicked, pooled, and packed into 10 g portions, which were then vacuum packed and subjected to a mild pasteurization treatment at 70 °C for 2 min in a water bath (GRANT Y28, Grant Instruments, Cambridgeshire, UK). The packs were then stored at 4 °C with sampling at time *t* = 0, 1, 4, 6, 8, 10, 14, 21, and 28 days. 

### 2.2. Microbiological Analysis

On each sampling day, 3 × 10 gram samples of each treatment were aseptically taken, diluted tenfold with maximum recovery diluent (MRD, Oxoid Ltd., Hampshire, UK), and homogenised for 1 min in a stomacher (Starblender LB400, VWR, Dublin, Ireland). A ten-fold dilution series was then prepared in MRD, and agar plates were inoculated by either a spread or pour plate technique. Total viable mesophilic counts were determined using plate count agar (Oxoid, CM0325) incubated at 30 °C for 72 h. Total enterobacteriaceae counts were obtained using violet red bile glucose agar (Oxoid, CM0485) incubated at 37 °C for 24 h. *Pseudomonas* spp. was determined using Pseudomonas agar base (Oxoid, CM0559) with Cephalothin-Sodium Fusidate-Cetrimide (CFC) supplement (Oxoid, SR103) incubated at 30 °C for 48 h. *Clostridium* spp. were grown on reinforced Clostridial agar (Oxoid, CM0151) at 30 °C for 72 h. Lactic acid bacteria (LAB) were grown on deMan Rogosa Sharpe (MRS, Oxoid, CM0361) agar at 30 °C for 72 h. Hydrogen disulphide producing bacteria were determined using Iron Lyngby agar, prepared as per NMKL at 25 °C for 72 h [19]. The shelf-life was considered over when the total viable count (TVC) reached 5–6 log_10_ cfu/g [20].

### 2.3. Measuring pH, a_w_ and WHC

The pH was measured at room temperature on undiluted crab meat samples using a surface electrode (Eutech Instruments pH5+ pH meter). The available water was determined at room temperature on undiluted crab meat samples using a water activity meter (Deacagon AquaLab LITE, Alton, UK). Water holding capacity (WHC) was determined using a centrifuge (eppendorf S810R, eppendorf, Wien, Austria) as per the method described by Zapata et al. [21].

### 2.4. Statistical Analysis

This experiment (5 crabs × 6 treatments) was undertaken on 3 separate occasions (90 crabs in total) and the data presented as a mean at each sampling time. For cook loss data, the means obtained with each treatment were compared with that obtained for the control using the unpaired *t*-test. A similar statistical method with *p* < 0.05 was used to compare water holding capacity. The mean bacterial counts, (within a given treatment and sampling time) were subjected to analysis using negative binomial distribution with a log link, performed in GENSTAT by Anova version 14.1 (VSN International Ltd., Hemel, Hempstead, UK).

## 3. Results 

Immediately after soaking and cooking, the cook loss was affected by the incorporation of functional ingredients, as shown in Table 1. Control samples averaged 11.1% cook loss and ascorbic acid treated samples showed 8.0% loss. However, this was significantly reduced to 3.5% (*p* < 0.025), 4.7% (*p* < 0.01), 5.8% (*p* < 0.05) and 2.3% (*p* < 0.01) with sodium caseinate, potato starch, sodium caseinate plus ascorbic acid, and potato starch plus ascorbic acid, respectively, as compared to water (control 1).

The pH of untreated crab meat ranged from 7.7 to 8.3 during storage at 4 °C. Similarly, samples treated with functional ingredients and preservatives also achieved a similar pH range of between 7.6 and 8.4. The a_w_ ranged from 0.96 to 0.99, regardless of treatment. Interestingly, there was no statistical differences (*p* > 0.05) in the water holding capacity of treated and untreated crab samples, which ranged from 73% to 82%. 

**Figure 1:**
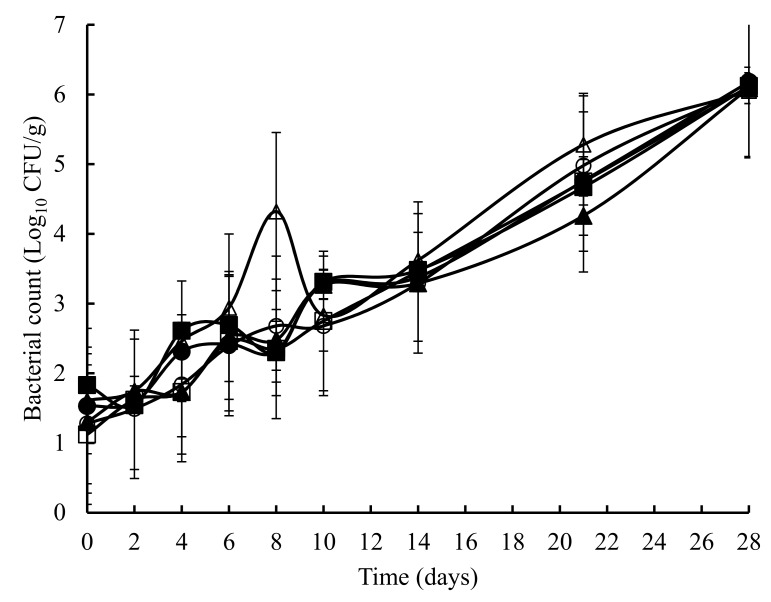
The total viable count (mesophilic) in crab meat samples stored at 4 °C with the following treatments: SDW (■), Ascorbic Acid (●), Casein (▲), Casein and Ascorbic Acid (□), Potato Starch (o) and Potato Starch & Ascorbic Acid (∆). The error bars represent 1 standard deviation.

**Figure 2:**
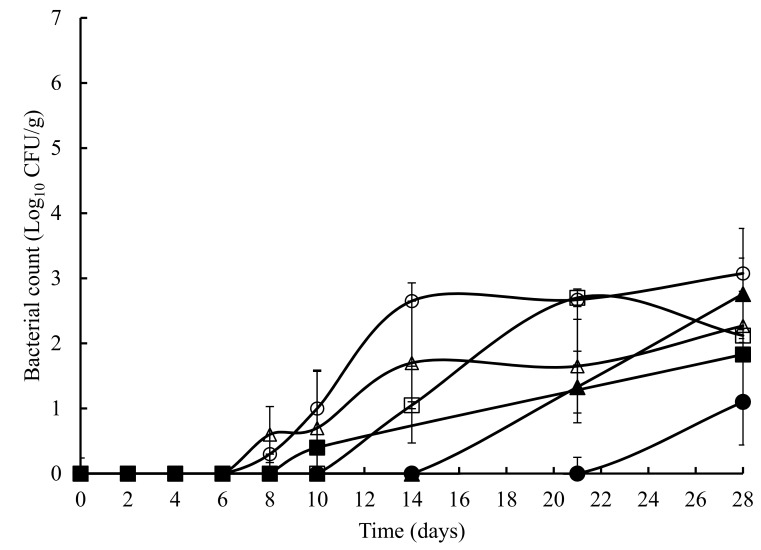
The total Enterobacteriaceae count in crab meat samples stored at 4 °C with the following treatments: SDW (■), Ascorbic Acid (●), Casein (▲), Casein & Ascorbic Acid (□), Potato Starch (o), and Potato Starch & Ascorbic Acid (∆). The error bars represent 1 standard deviation.

**Figure 3:**
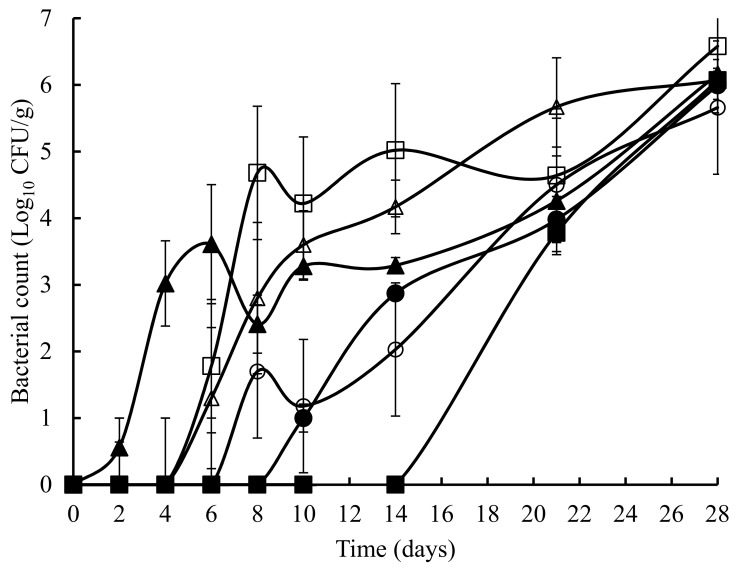
*Pseudomonas* spp. in crab meat samples stored at 4 °C with the following treatments: SDW (■), Ascorbic Acid (●), Casein (▲), Casein & Ascorbic Acid (□), Potato Starch (o), and Potato Starch and Ascorbic Acid (∆). The error bars represent 1 standard deviation.

**Figure 4:**
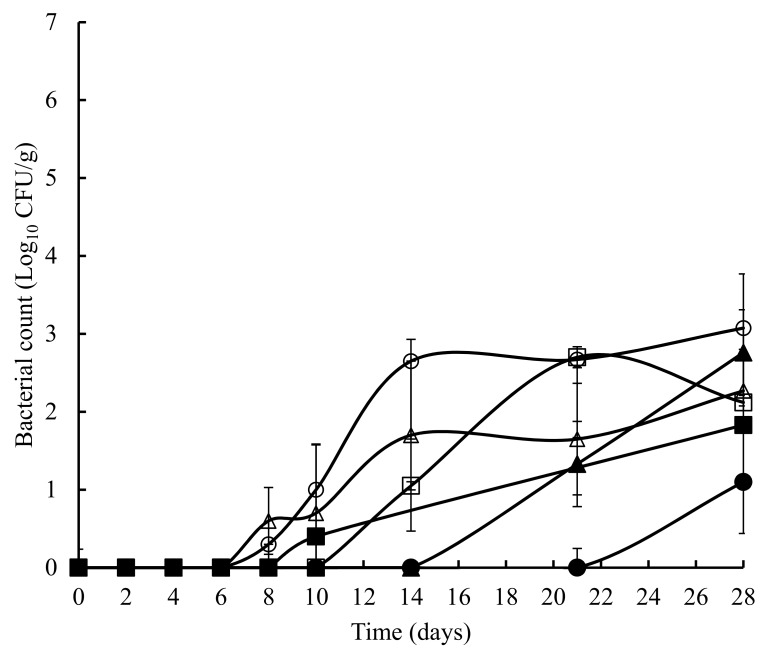
Hydrogen disulphide (H2S) producing bacteria in crab meat samples stored at 4 °C with the following treatments: SDW (■), Ascorbic Acid (●), Casein (▲), Casein and Ascorbic Acid (□), Potato Starch (o), and Potato Starch and Ascorbic Acid (∆). The error bars represent 1 standard deviation.

**Figure 5:**
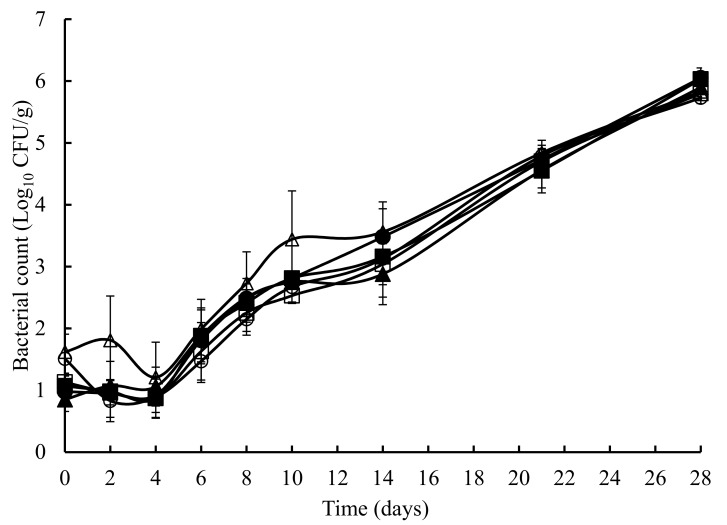
*Clostridium* spp. in crab meat samples stored at 4 °C with the following treatments: SDW (■), Ascorbic Acid (●), Casein (▲), Casein and Ascorbic Acid (□), Potato Starch (o), and Potato Starch and Ascorbic Acid (∆). The error bars represent 1 standard deviation.

**Figure 6:**
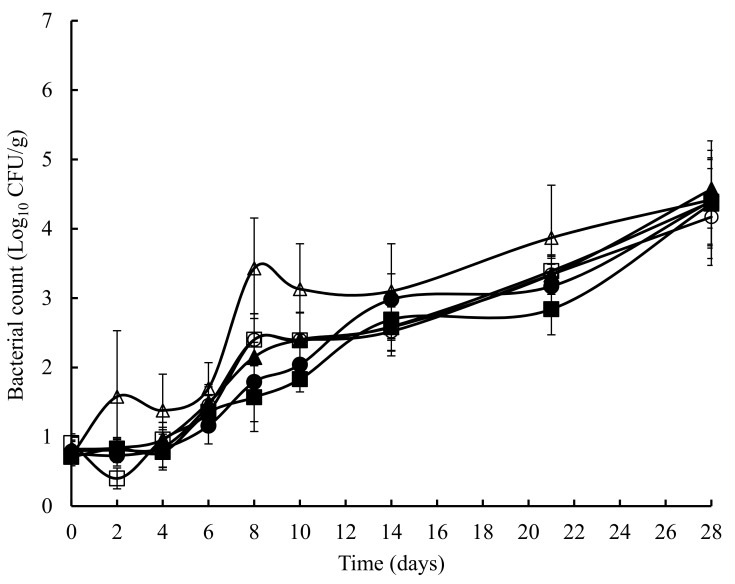
Lactic Acid Bacteria in crab meat samples stored at 4 °C with the following treatments: SDW (■), Ascorbic Acid (●), Casein (▲), Casein and Ascorbic Acid (□), Potato Starch (o), and Potato Starch and Ascorbic Acid (∆). The error bars represent 1 standard deviation.

The TVC obtained on untreated and treated crab meat, over a 28 days storage at 4 °C, is presented in Figure 1. The initial TVC ranged from 1.2 to 1.8 log_10_ cfu/g and with the exception of the potato starch and ascorbic acid samples at 8 days, statistically (*p* > 0.05) similar counts were obtained throughout the experiment. Overall, a similar pattern (with the odd time specific exception) was observed with TEC (Figure 2), *Pseudomonas* spp. (Figure 3), hydrogen disulphide producing bacteria (Figure 4), *Clostridium* spp. (Figure 5), and lactic acid bacteria (LAB) (Figure 6). TEC, *Pseudomonas* spp. and hydrogen disulphide producing bacteria, although not detected initially, reached a final count of 4.2, 6.0, and 1.8 log_10_ cfu/g, respectively, after 28 days at 4 °C. The initial counts for *Clostridium* spp. and lactic acid bacteria (LAB) were 1.0 and 0.76 log_10_ cfu/g, respectively, which increased to 6.0 and 4.4 log_10_ cfu/g, respectively, after 28 days at 4 °C.

## 4. Discussion and Conclusions 

To the best of our knowledge, there have been no studies that investigate the potential of water binders to decrease the cook loss of Irish brown crab during processing nor have the effect on these ingredients on bacterial growth and shelf-life been investigated during chilled storage. Crab processing includes a pre-soak stage in water at ambient temperature for approximately 2 h followed by boiled at 100 °C for 20 min. In live crabs, the shell restricts solution penetration into the musculature beneath. In dead crabs, the soaking stage facilitates the dissolution of protein tissues in the tegumental gland ducts, facilitating direct contact between the solution and the meat [22]. Thus, a multitude of commonly used recipes add seasoning to the soak water to enhance the flavor of the meat obtained later in the process. Boiling may also soften the crabs shell facilitating the transfer of ingredients into the meat. However, the process of getting specific ingredients into the crab could be improved through the application of high pressure processing that has also been shown to contribute to higher yield [23].

In our study, the different ingredients were added during the soak and boiling stages. Cook loss was decreased from approximately 11% to 3.5% (*p* < 0.025), 4.7% (*p* < 0.01), 5.8% (*p* < 0.05), and 2.3% (*p* < 0.01) when sodium caseinate, potato starch, sodium caseinate plus ascorbic acid, and potato starch plus ascorbic acid, respectively, were added in the pre-soaked and boiled stages of processing. This was not unexpected, as sodium caseinate and potato starch have previously been reported to reduce cook loss by 5–7% in other meat products [24,25,26]. Sodium caseinate acts as a surface-active material capable of binding meat protein and fat globules [27]. In processed meat, it is also widely used to as a water binding material [26,28]. All of these functions increase yield and decrease shrinkage, while contributing high quality protein [29]. Starch also binds water, through the interaction of the oxygen atoms in its constituent glucan units. However, starch also forms a complex with meat proteins at temperatures above the gelation temperature (50–70 °C) of the starch, which may have reduced the protein loss during the crab boiling stage of processing [29,30]. Maintaining yield is important in terms of commercial value but also because an increasing weight loss concentrates any chemical contaminants present in the crab [31] causing an increased risk to human health [32]. However, the yield gains reported in this study may still not be sufficient to ensure compliance with current EU limits for specific contaminants such as cadmium [33].

Many bacteria can use sodium caseinate and potato starch as an energy source with the latter also providing amino acids, essential for bacterial growth and multiplication. Thus, it was important to investigate the potential effect of sodium caseinate and potato starch on bacterial growth and shelf-life. Moreover, the pH of all samples was in the range of 7.7 to 8.3, which is favorable for the proliferation of bacteria. Interestingly, our data showed similar growth, and hence shelf-life, regardless of the addition, or otherwise, of sodium caseinate or potato starch, with or without ascorbic acid. Few other studies have examined the impact of functional ingredients on shelf-life and the few that have done so are not directly comparable with our work. Zargar and Yeganeh [34], for example, reported no reduction in the shelf-life of rainbow trout treated with sodium caseinate in combination with *Zataria multiflora* essential oil but this may have been due to the antimicrobial actions of the latter. Interestingly, our study also suggests that ascorbic acid, a commonly used food preservative, did not increase shelf-life. Kilic and Oztan [18] made a similar finding in their investigation of ascorbic acid as a preservative in smoked fish.

Based on our observations, it was concluded that, subject to sensory evaluation and validation under commercial conditions, sodium caseinate or potato starch could be used to substantially increase the yield and hence commercial value of crab meat, without adversely affecting shelf-life.

## Figures and Tables

**Table 1:** Mean cook loss (%) of whole crabs when sodium caseinate and potato starch with and without ascorbic acid, were incorporated into the processing stages.

	Mean	S.E.M ^1^ (*n* = 5)	Significance
Water (control 1)	11.1	2.21	
AA ^2^ (control 2)	8.0	4.86	NS^5^
SC ^3^	3.5	3.71	*p* < 0.025
PS ^4^	4.7	4.55	*p* < 0.01
SC + AA	5.8	3.26	*p* < 0.05
PS + AA	2.3	3.52	*p* < 0.01

^1^ Standard Error of Mean; ^2^ Ascorbic Acid; ^3^ Sodium Caseinate; ^4^ Potato Starch, ^5^ Not significant
